# Optimization of Encapsulation Core–Shell Structure to Preserve Polyphenols in Soy Protein—Polysaccharide Co-Dried Complexes

**DOI:** 10.3390/molecules30050978

**Published:** 2025-02-20

**Authors:** Xinyue Zheng, Xiaofang Chu, Hongyang Pan

**Affiliations:** 1 Engineer Faculty, The University of Sydney, Sydney 2008, Australia; eudoragogogo@gmail.com; 2 Institute of Botany, Jiangsu Province and Chinese Academy of Sciences, Nanjing 210014, China; cxf1002@163.com; 3 State Key Laboratory of Food Science and Resources, Jiangnan University, Wuxi 214122, China; 4 Analysis and Testing Center, Jiangnan University, Wuxi 214122, China

**Keywords:** extra virgin olive oil (EVOO), soy protein isolate (SPI), maltodextrin (MD), propylene glycol alginate (PGA), encapsulation, core–shell structure, food-grade delivery system

## Abstract

Polyphenols from extra virgin olive oil (EVOO) are bioactive compounds with significant antioxidant properties, but their instability necessitates effective encapsulation for enhanced stability and controlled release. This study prepared water-in-oil-in-water (W1/O/W2) emulsions to encapsulate EVOO using a two-step emulsification technique with varying concentrations of soy protein isolate (SPI) (0–10% *w*/*w*), maltodextrin (MD) (0–20% *w*/*w*), and propylene glycol alginate (PGA) (0–0.5% *w*/*w*). A three-factor central composite design (CCD) combined with response surface methodology (RSM) was employed to establish 20 W1/O/W2 emulsions to analyze the effects of the formulation on emulsion properties. Additionally, the effects of different pH levels on emulsion stability were investigated. The results showed that the ratios of SPI, MD, and PGA significantly influenced particle size distribution, stability, and encapsulation efficiency. PGA enhanced the rigidity of the interfacial membrane, forming stable core–shell structures and reducing EVOO release. The optimal formulation (7.887% SPI, 15.774% MD, 0.395% PGA) achieved superior encapsulation efficiency (97.66%), long-term stability, and viscosity below 300 mPa·s. Cryo-TEM analysis confirmed the formation of core–shell structures, while Zeta potential measurements indicated smaller particle sizes and enhanced stability at pH 11. This optimized W1/O/W2 emulsion system offers a promising food-grade delivery platform for hydrophobic bioactive compounds, enabling enhanced stability and controlled release of EVOO polyphenols for applications in functional foods, nutraceuticals, and other industries.

## 1. Introduction

Extra virgin olive oil (EVOO), mechanically and physically extracted from olive fruit (*Olea europaea* L.) at low temperatures [1], is favored for its oxidative stability and unique aroma compared to common edible oils such as sunflower and soybean oils. EVOO is also renowned for its health benefits, attributed to bioactive compounds such as polyphenols, oleocanthal, and oleuropein [2,3]. However, the sensitivity of EVOO polyphenols to environmental factors necessitates effective encapsulation techniques to improve their stability and bioavailability [4].

Double emulsions, particularly water-in-oil-in-water (W/O/W) emulsions, offer a promising approach for encapsulating phenolic compounds by protecting the oil phase from external degradation [5,6,7]. The stability and controlled release of active ingredients in these emulsions are influenced by emulsifiers, structural properties of biopolymers, and homogenization conditions [8].

Processing steps such as emulsion preparation, storage conditions, and spray drying enhance the stability of phenols compounds in double emulsion products, demonstrating their effectiveness in encapsulating sensitive bioactive ingredients [9,10]. Variations in coating materials and phenolic properties lead to different interactions and preparation methods, with stable formulations achieving high encapsulation efficiency and effective protection for controlled release in food systems [11]. Embedding active ingredients in W/O/W emulsions significantly improves their physical and chemical stability, allowing them to resist environmental stress and release gradually, thereby enhancing bioavailability [12].

Multi-layer emulsions utilize synergistic interactions between biopolymers to stabilize the interfacial emulsification system [13]. Primary EVOO droplets are formed by homogenizing the water phase, EVOO phase, and an emulsifier, followed by the adsorption of biopolymers to create a secondary layer that protects active substances [12]. These emulsions can be designed to deliver active ingredients to target sites and release them gradually [14]. Common edible coating materials include whey protein, gelatin, soy protein isolate (SPI), gum arabic, maltodextrin (MD), gum, and emulsifying starch [15,16]. Recent advancements highlight blending proteins and polysaccharides under controlled conditions, such as adjusting temperature and pH, to enhance stability [17].

SPI, an eco-friendly alternative to animal proteins, offers good emulsion stability and volatile component retention but exhibits poor mechanical properties when used alone [18]. To enhance its performance as a coating material, SPI can be blended with MD or propylene glycol alginate (PGA) to create binary blends or combined with both to form ternary complexes [19,20]. MD, when used alone, extends the shelf life of rosemary essential oil [21], but its application in microencapsulation is limited by poor emulsifying properties and low retention of flavor compounds [22]. PGA, a hydrophilic colloidal carbohydrate, is often blended with whey protein or gelatin. Its versatile properties, including stability, suspension capability, film forming, gelling, and emulsifying stability, make it a promising candidate for biopolymer films and coating materials [23].

Despite multiple studies on using proteins, polysaccharides, and their blends in double emulsions, there remains a knowledge gap in optimizing EVOO-based double emulsions to enhance phenolic preservation and system stability [24]. This study seeks to address this gap by co-dry blending SPI, MD, and PGA to develop a stable W/O/W emulsion system enriched with EVOO phenolics. Using response surface methodology (RSM), we optimized material proportions to achieve maximum encapsulation efficiency (EE%) and analyzed the impact of pH on emulsion stability during preparation. This research aims to advance the practical application of EVOO emulsions in functional foods and nutraceuticals.

## 2. Results and Discussion

### 2.1. Particle Size Distribution of Complex W1/O/W2 Emulsions

**Figure 1:**
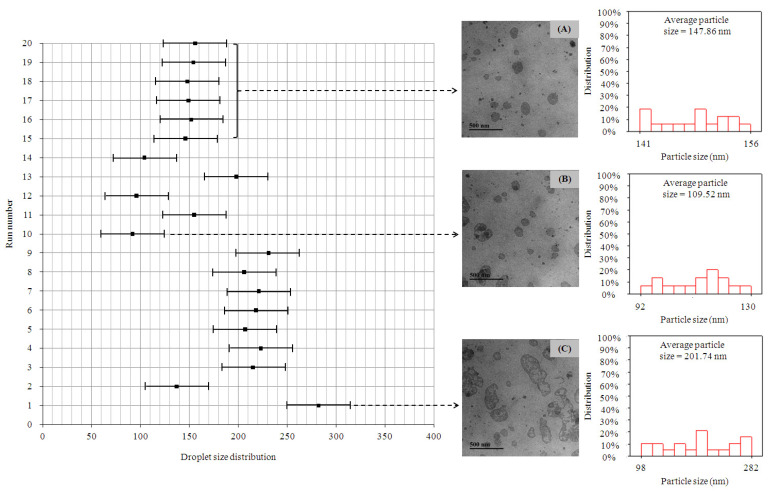
Cryo-transmission electron microscope images and size distribution of the complex W1/O/W2 emulsions: (**A**) 5% (*w*/*w*) SPI + 10% (*w*/*w*) MD + 0.25% (*w*/*w*) PGA (run numbers 15–20); (**B**) 10% (*w*/*w*) SPI + 10% (*w*/*w*) MD + 0.25% (*w*/*w*) PGA (run number 10); (**C**) 2.113% (*w*/*w*) SPI + 4.227% (*w*/*w*) MD + 0.106% (*w*/*w*) PGA (run number 1). W1/O/W2: water-in-oil-in-water, SPI: soy protein isolate, MD: maltodextrin, PGA: propylene glycol alginate.

The particle size distribution is a crucial indicator of the stability of W1/O/W2 emulsions after storage for 24 h at 25 °C. As shown in Figure 1, the particle sizes were classified into three distinct groups: small particles (approximately 92–130 nm), medium-sized particles (approximately 141–156 nm), and large particles (approximately 198–282 nm). To assess the effects of varying proportions and concentrations of soy protein isolate (SPI), maltodextrin (MD), and propylene glycol alginate (PGA), the average particle size was used for comparison. Previous studies have demonstrated that the addition of polysaccharides significantly affects both particle size and its distribution [25]. Microstructure analysis revealed that multiple W/O droplets in water formed a coated emulsion structure (Figure 1). The optimal particle size distribution was observed in run number 10, which utilized a combination of 10% *w*/*w* SPI, 10% *w*/*w* MD, and 0.25% *w*/*w* PGA as wall materials. The variations in particle size distribution were attributed to the different proportions and concentrations of polysaccharides, which interacted in distinct ways, influencing the overall size and stability of the emulsions [26].

### 2.2. Gelling Properties of Complex W1/O/W2 Emulsions

Under alkaline conditions (pH = 9.0), SPI and PGA are anionic polymers, which played a significant role in the aggregation process, acting as effective reactants. When conditions are suitable, they can serve as embedding or encapsulating materials, forming stable colloidal structures [27]. MD, due to its high solubility, was considered an efficient embedding material and could result in lower viscosity at higher concentrations in W1/O/W2 emulsions. However, MD had the drawback of poor interfacial properties, which are crucial for enhancing embedding efficiency. To address this, MD is often combined with materials that have strong interfacial properties [28]. Studies on the properties of MD suggested that MD with a dextrose equivalent (DE) between 10 and 20 is ideal for use as a coating material, owing to its strong embedding efficiency [29].

PGA is well known for its strong gelling properties, which help stabilize emulsions by promoting flocculation and aggregation [30]. Key indicators of emulsion instability include the particle size distribution of the droplets, and the emulsifying index. According to the emulsifying index, emulsions with smaller particles tend to be more stable [31]. Furthermore, the overall stability of the emulsion directly impacts the stability of the coating system and, consequently, the effectiveness of the encapsulated bioactive ingredients.

### 2.3. Effect of the Ratio Between Biopolymers and Their Concentration on Properties of Complex W1/O/W2 Emulsions

An increase in the proportion of PGA in the blend contributed significantly to an increase in encapsulation efficiency of the W1/O/W2 emulsion (Table 1). The 0.395% (*w*/*w*) PGA provided superior protection to the EVOO compared to PGA individually, as well as its combination with MD or SPI. Considering the interactions between polysaccharides, SPI and MD could have a larger influence compared to other materials, and a greater synergistic effect was observed between SPI and MD. As an expansive agent, MD could assist the gelling reaction of SPI. As shown in Table 1, encapsulation efficiency varied between 75.13% and 97.66%, influenced by the formulation of the complex W1/O/W2 emulsions. Co-drying blending of coating materials increased the thickness of the coating layer and also reduced phenol loss during storage. This finding aligns with previous reports on the influence of complexation colloid properties on embedding processes in multiple emulsion systems and long-term preservation [32,33]. These results are consistent with previous research on microencapsulation of folic acid [25].

### 2.4. Optimization of Complex W1/O/W2 Emulsions Using Response Surface Analysis

RSM helps to reduce the number of experimental trials required through statistical predictions; it also facilitates studying interactions and effects between multiple factors [34]. The values for encapsulation efficiency, creaming index, and viscosity for each trial are provided in Table 1.

CCD results are presented in Table 2. Statistical analysis showed no evidence of “lack of fit” because *p*-values for all parameters were higher than 0.05 (Table 2). Moreover, coefficients of determination ranged from 0.865 to 0.981, confirming model desirability for elucidating variable relationships and allowing acceptable fitness of proposed models to experimental results. High R^2^ (adj) values (from 0.680 to 0.955) demonstrate that non-significant terms have not been included in the model. Creaming stability, viscosity, and encapsulation efficiency values were obtained after storage at 25 °C for 24 h. These variables yielded the highest R^2^ and adjusted R^2^ for each response variable studied. All proposed models were significant for all variables (creaming index (*p* < 0.0001), viscosity (*p* < 0.0001), and encapsulation efficiency (*p* = 0.2001)), as shown in Table 2. The quadratic model’s confidence level was confirmed by R^2^; the total model was highly significant with corresponding coefficients R^2^ = 0.680–0.955, indicating that 68.0–95.5% of response variability could be explained by the model. For multiple emulsions, all interaction effects of protein and polysaccharide matrices had insignificant effects (*p* > 0.05) on creaming stability and encapsulation efficiency. As shown in Table 2, considering the highest coefficient of main linear effects, PGA had a significant effect (*p* < 0.05) on encapsulation efficiency after storage at 25 °C for 24 h. Similar results have been reported by others too [35]. The gel formed after adding PGA reduced the release of internal phase EVOO from the emulsion to the external phase. As a hydrophilic colloid carbohydrate, PGA demonstrated unique colloidal properties when complexed with SPI, including stability, suspension capability, film-forming ability, gelling property, and emulsifying stability, which had a synergistic effect when complexed with SPI in a W1/O/W2 emulsion system, thus becoming a potential biopolymer film material or coating component with superior performance [36]. The quadratic model of SPI showed a significant (*p* < 0.05) but variable effect on response variables.

**Figure 2:**
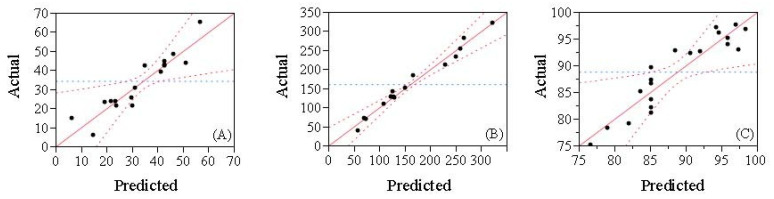
Predicted values and the experimental values of (**A**) creaming index (%), (**B**) viscosity (η, mPa), and (**C**) encapsulation efficiency (EE, %) of 7.887% (*w*/*w*) SPI + 15.774% (*w*/*w*) MD + 0.395% (*w*/*w*) PGA complex W1/O/W2 emulsions.

The predicted values were calculated using model-fitting techniques; fitting data to different models (linear, two-factorial, quadratic, and cubic), the quadratic polynomial model was most suitable according to ANOVA results. A strong relationship between predicted and experimental values was evident, as shown in Figure 2. The study’s purpose using response surface modeling was to find an optimal region for response variables and determine correlations between three independent variables and response variables. As shown in Figure 2, a formula containing 7.887% (*w*/*w*) SPI, 15.774% (*w*/*w*) MD, and 0.395% (*w*/*w*) PGA was optimal, yielding the best encapsulation efficiency and stability in a favorable viscosity region (<300 mPa).

Comparing experimental values with those predicted by response regression modeling verified equation adequacy and validated the model. No significant difference existed between predicted and experimental values (*p* > 0.05).

### 2.5. Effect of pH on Stability of Complex W1/O/W2 Emulsions

**Figure 3:**
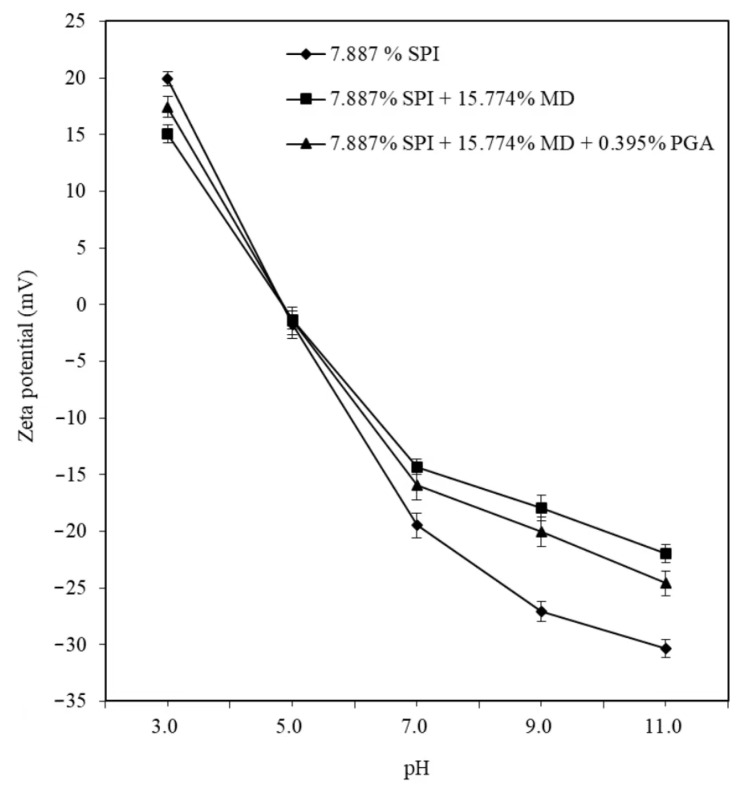
Comparison of Zeta potentials of bare 7.887% (*w*/*w*) SPI, 7.887% (*w*/*w*) SPI + 15.774% (*w*/*w*) MD, and 7.887% (*w*/*w*) SPI + 15.774% (*w*/*w*) MD + 0.395% (*w*/*w*) PGA complex W1/O/W2 emulsions at pH values of 3.0, 5.0, 7.0, 9.0, and 11.0. Error bars are standard deviations from duplicate experiments, and each were measured three times.

Zeta potential measurements revealed pI and pKa values for single SPI, MD, and PGA; the pI for single SPI was 4.7, while pKa values for single MD and PGA were 6.8 and 3.5, respectively, consistent with literature reports (SPI pI = 4.5; MD pKa = 6.8–6.9; PGA pKa = 3.38–3.65) [37,38,39]. The influence of pH on the Zeta potentials of SPI/MD/PGA complex W1/O/W2 emulsions is depicted in Figure 3.

The absolute Zeta potential value of co-blended 7.887% (*w*/*w*) SPI + 15.774% (*w*/*w*) MD emulsions approached zero across a pH range of 3.0 to 11.0, indicating that in complex W1/O/W2 emulsions, aggregation of SPI and MD created steric hindrance from polysaccharide aggregation on SPI droplet surfaces, inhibiting droplet movement and shielding surface charges [40].

**Figure 4:**
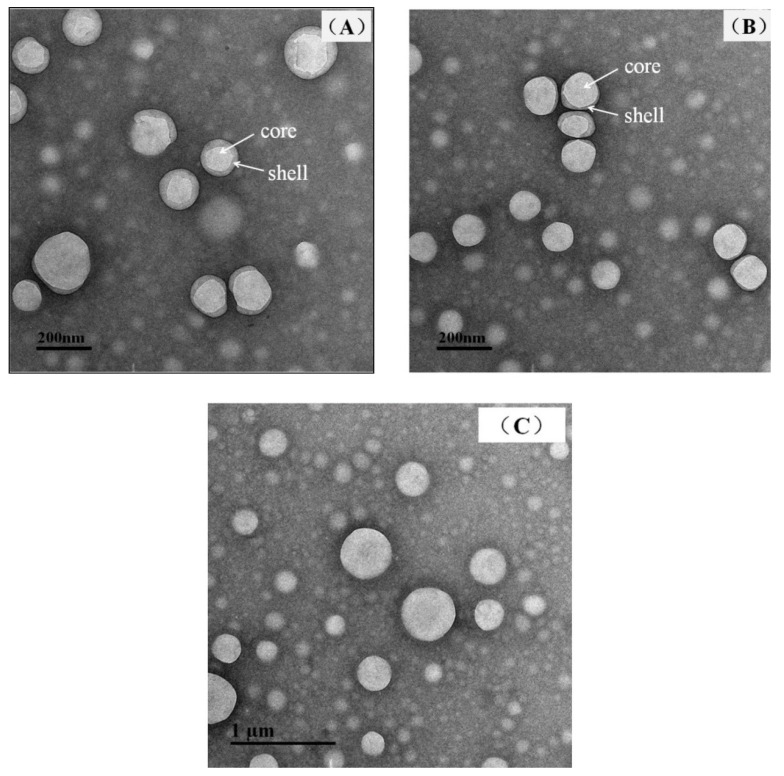
The core–shell structures of complex W1/O/W2 emulsions: (**A**) 7.887% (*w*/*w*) SPI + 15.774% (*w*/*w*) MD + 0.395% (*w*/*w*) PGA, pH = 9; (**B**) 7.887% (*w*/*w*) SPI + 15.774% (*w*/*w*) MD + 0.395% (*w*/*w*) PGA, pH = 11; (**C**) 7.887% (*w*/*w*) SPI + 15.774% (*w*/*w*) MD, pH = 9.

Moreover, within the designated pH range, the absolute Zeta potential values of 7.887% (*w*/*w*) SPI + 15.774% (*w*/*w*) MD + 0.395% (*w*/*w*) PGA complex W1/O/W2 emulsions, obtained by the co-drying method, were higher than those of the 7.887% (*w*/*w*) SPI + 15.774% (*w*/*w*) MD emulsion systems. This increase was likely due to the core–shell structure (Figure 4) of the 7.887% (*w*/*w*) SPI + 15.774% (*w*/*w*) MD + 0.395% (*w*/*w*) PGA system formed during the co-drying process. The interfacial membrane rigidity was believed to have increased by PGA addition, which resulted in a more stable gelling structure and a higher absolute Zeta potential value. These results can be attributed to PGA’s ability to form gels, thereby reducing the release of phenolic components from the inner to the outer phase of the emulsion [41]. The microstructures of complex W1/O/W2 emulsion particles observed by Cryo-TEM are shown in Figure 4. Cryo-TEM images revealed that the particles exhibited a spherical structure. MD and PGA, with their hydrophilic chains, displayed distinct contrast from SPI and were uniformly dispersed on the particle surfaces. The core–shell structures of W1/O/W2 emulsions varied with pH. TEM analysis determined that the average particle size of the core–shell structure was 194.03 nm at pH 9 and 123.72 nm at pH 11, further illustrating that a more stable structure correlated with smaller particles at pH 11 (7.887% (*w*/*w*) SPI + 15.774% (*w*/*w*) MD + 0.395% (*w*/*w*) PGA).

## 3. Materials and Methods

### 3.1. Materials and Reagents

The EVOO used as an active material (ρ = 905 kg/m^3^, μ = 0.080 Pa·s at 25 °C) was sourced from Textron Inc. (Shanghai, China), originating from Spain. Soy protein isolate (SPI) with a protein content of 94.50 ± 0.06g/100g (dry basis) was provided by Jiangnan University (Wuxi, China). Maltodextrin (MD) with a dextrose equivalent (DE) of 19 and propylene glycol alginate (PGA) with a relative molecular mass of 234.32 were supplied by Fisher Sci. Ltd. (Shanghai, China) as wall materials for this study. Tween 20 and polyglyceryl-10 polyricinoleate were obtained from Merck (Darmstadt, Germany) and used as stabilizers for the double emulsions.

### 3.2. Preparation of SPI/MD/PGA Complexation Powders

The SPI/MD/PGA complexation solutions were prepared according to the method developed by Pan [42]. The concentrations of SPI and the mass ratios of MD to PGA in the co-blended system were determined according to Table 3. To ensure the emulsion viscosity was suitable for spray drying, the formulation was adjusted. Using a handheld laboratory homogenizer (Model T25, Janke Kunkel, IKA Labortechnik, Shanghai, China), the W1/O emulsion was incorporated into the outer phase (W2) at 10,000 rpm for 2 min to prepare the final double emulsion. The prepared emulsions were stored at 25 °C for 24 h before stability analysis, apparent viscosity measurement, and Cryo-TEM imaging. Encapsulation efficiency was also determined after the 24 h storage period.

### 3.3. Preparation of SPI/MD/PGA Co-Blended W1/O/W2 Emulsions

A modified two-step emulsification method was employed to prepare the W/O/W double emulsion, as described by Xie et al. [43]. To create the W/O emulsion, EVOO (oil phase) was heated to 60 °C, then mixed with the hydrophobic surfactant polyglycerol polyricinoleate (hydrophilic–lipophilic balance of 0.6), and stirred for 15 min using a magnetic stirrer. The internal aqueous phase (W1), composed of 3% gelatin, 2% NaCl, 0.16% *w*/*w* polyglyceryl-10 polyricinoleate, and deionized water, was also heated to 60 °C. The W/O emulsion was formed by adding the water phase drop-wise into the oil phase. After adding the NaCl and EVOO solutions, the temperature was maintained at 60 °C for 5 min. The composition of the outer phase (W2) is detailed in Table 3. To optimize the process, a three-factor central composite design (CCD) was used to prepare 20 samples with varying concentrations of SPI (0–10% *w*/*w*), MD (0–20% *w*/*w*), and PGA (0–0.5% *w*/*w*). The concentration ranges were selected based on prior research findings.

### 3.4. Determination of Encapsulation Efficiency of SPI/MD/PGA Co-Blended W1/O/W2 Emulsions

The encapsulation process of SPI/MD/PGA co-blended W1/O/W2 emulsions was monitored by measuring the encapsulation efficiency (EE%). EE% was calculated as the percentage of EVOO encapsulated within the microcapsules relative to the total EVOO content in the emulsion:

(1) $$EE\left(\%\right)=\frac{Encapsulated\;EVOO\;load-EVOO\;load\;on\;the\;surface}{Encapsulated\;EVOO\;load}\times 100\%$$

The encapsulated EVOO load was determined using the method described in a previous study [44]. To assess the EVOO load on the surface, n-hexane extraction was carried out. A 5 g sample of the W1/O/W2 emulsion was placed in a flask with 10 mL of n-hexane, gently shaken, and then filtered through filter paper. The same method was applied to the SPI/MD/PGA co-blended W1/O/W2 emulsions for EVOO load determination.

### 3.5. Determination of Stability

Samples of 10 g of W1/O/W2 emulsion were transferred into test tubes (internal diameter 13 mm, height 150 mm), which were then tightly sealed with plastic caps. For thermal stability testing, the test tubes containing the W1/O/W2 emulsions were stored at 25 °C for 24 h in a water bath. Then, they were centrifuged at 200 rpm for 10 min. The stability of the emulsions after storage for 10 h (creaming stability) was measured by visual observation based on phase separation and expressed as a percentage of initial sample height [45]. The measurements were performed in triplicate. The total height of the emulsion (HE) and the height of the serum layer (HS) were measured. The extent of creaming was quantified using the creaming index (%), calculated as follows:

$$Creaming\;index\left(\%\right)=\frac{HS}{HE}\times 100\%$$

The creaming index provided indirect information on the extent of droplet aggregation in the complex W1/O/W2 emulsions; a higher creaming index indicated faster creaming and larger particle size.

### 3.6. Measurement of Viscosity

The viscosities of the prepared co-blended W1/O/W2 emulsions were measured at 25 °C using a Brookfield DV-II+ Pro LV viscometer (Brookfield Engineering Laboratories, Shanghai, China) with concentric cylinder geometry. Viscosity (η) values were recorded in centipoise (mPa·s).

### 3.7. Cryo-Transmission Electron Microscope (Cryo-TEM) Imaging of Emulsions

A laboratory-built humidity-controlled room, maintained at 50% relative humidity (RH) and 25 °C, was used to prepare samples for Cryo-TEM imaging. A 5 μL aliquot of emulsion was placed onto a grid covered with lacey carbon film (200 mesh, hole width 0.09 mm). After excess sample was removed by blotting with filter paper, the sample grid was vitrified by rapid plunging into liquid ethane at −180 °C to pre-cool, followed by freezing the film to a thickness of approximately 200 nm. The prepared frozen grids were stored in liquid nitrogen at −196 °C before being transferred to a Gatan 626 cryo-holder. Cryo-TEM images were captured using a JEOL JEM-2100 TEM (200 kV, Tokyo, Japan) under low-dose conditions, with a charge-coupled device (CCD) imaging system and DM 3.5 software. Size distribution and average size analysis of the emulsions were conducted using Nano Measurer 1.2.5 software (Shanghai, China).

### 3.8. Determination of Zeta Potential

The Zeta potential of the W1/O/W2 emulsions was measured using a Zetasizer Nano ZS (Malvern Instruments, Malvern, UK) at 25 °C. Each reported value represents the mean and standard deviation of six measurements, with two replications conducted for each sample.

### 3.9. Experimental Design and Statistical Analysis

Response surface methodology (RSM) with a central composite design (CCD) was employed for analysis. The independent variables (SPI content, MD content, and PGA content) were studied, with stability, encapsulation efficiency, droplet size distribution, and apparent viscosity of the double emulsion serving as the response variables. A total of 20 experimental trials were conducted, with the first 14 organized in a factorial design and six center points included to assess the reproducibility of the method. After performing the experiments, a polynomial model was used to predict linear, quadratic, and interaction effects of the independent variables in RSM:

*Y* = *β*_0_ + *β*_1_*X*_1_ + *β*_2_*X*_2_ + *β*_3_*X*_3_ + *β*_12_*X*_1_*X*_2_ + *β*_13_*X*_1_*X*_3_ + *β*_23_*X*_2_*X*_3_ + *β*_11_*X*_1_^2^ + *β*_22_*X*_2_^2^ + *β*_33_*X*_3_^2^ (2)

where *Y* is the response variable; *β*_0_ is the constant term; *β*_1_, *β*_2_, and *β*_3_, are the linear coefficients; *β*_12_, *β*_13_, and *β*_23_, are the interaction coefficients; and *β*_11_, *β*_22_, and *β*_33_, are the quadratic coefficients.

The experimental design and data were analyzed using Design-Expert version 7.1.3 (State-Ease, Inc., Minneapolis MN, USA). Regression analysis of variance (ANOVA) was performed for the polynomial model equation using the same software. The fitness of the second-order equation was evaluated by the R^2^ value, and statistical significance was determined by the F-test. The significance of the regression coefficients was assessed through Student’s *t*-test [46,47].

## 4. Conclusions

This study explored the effects of varying proportions and concentrations of soy protein isolate (SPI), maltodextrin (MD), and propylene glycol alginate (PGA) on the particle size distribution, stability, and encapsulation efficiency of W1/O/W2 emulsions, providing insights into their key characteristics and optimal formulation. The research clarified the key characteristics and optimal conditions for these complex emulsions.

The particle size distribution of W1/O/W2 emulsions stored at 25 °C for 24 h showed three distinct size groups: small particles (92–130 nm), medium particles (141–156 nm), and large particles (198–282 nm). The addition of polysaccharides significantly influenced the particle size and distribution. The combination of SPI, MD, and PGA played a crucial role in forming stable encapsulating structures, with the formulation containing 10% (*w*/*w*) SPI, 10% (*w*/*w*) MD, and 0.25% (*w*/*w*) PGA (run number 10) achieving the optimal particle size distribution and stability. The addition of PGA significantly enhanced the rigidity and stability of the interfacial membrane, forming a more stable gel structure and reducing the release of internal phase components to the external phase. Furthermore, the synergistic interaction between SPI and MD improved emulsion stability, as MD’s expansive properties promoted SPI’s gelling reactions, while PGA’s strong gel-forming properties further enhanced the emulsions’ encapsulation performance.

The encapsulation efficiency of emulsions with different formulations ranged from 75.13% to 97.66%. Among the tested formulations, the optimal combination was found to be 7.887% (*w*/*w*) SPI, 15.774% (*w*/*w*) MD, and 0.395% (*w*/*w*) PGA, which achieved the best encapsulation efficiency and stability with viscosity maintained below 300 mPa·s. Model validation demonstrated no significant difference (*p* > 0.05) between predicted and experimental values, confirming the model’s reliability (R^2^ = 0.680–0.955). Cryo-TEM analysis confirmed the formation of core–shell structures, with synergistic interactions between SPI, MD, and PGA enhancing interfacial rigidity and gel stability. Zeta potential measurements further revealed that smaller particle sizes at pH 11 corresponded to more stable emulsions.

This research demonstrates the critical role of protein and polysaccharide combinations in enhancing the performance of W1/O/W2 emulsions for the encapsulation of bioactive compounds. The optimized formulation provides a stable and efficient delivery system for sensitive bioactive ingredients, such as polyphenols from extra virgin olive oil (EVOO), with potential applications in food, nutraceuticals, and other industries requiring controlled release systems.

## Figures and Tables

**Table 1:** Experimental values of response variables obtained from the central composite experiment design at 25 °C for 24 h.

Run Number	Independent Variables (%, *w*/*w*)			Response Variables		
	SPI (X_1_)	MD (X_2_)	PGA (X_3_)	Creaming Index (%) (Y_1_)	Viscosity (η, mPa) (Y_2_)	Encapsulation Efficiency (EE, %) (Y_3_)
1	2.113	4.227	0.106	65.52	40.60	78.32
2	7.887	4.227	0.106	21.42	183.43	92.64
3	2.113	15.774	0.106	25.68	127.81	92.88
4	7.887	15.774	0.106	23.89	321.89	94.06
5	2.113	4.226	0.395	30.68	127.35	92.37
6	7.887	4.227	0.395	23.36	69.01	93.01
7	2.113	15.774	0.395	15.09	212.71	95.24
8	7.887	15.774	0.395	6.20	283.04	97.66
9	0.000	10.000	0.250	43.69	142.22	79.12
10	10.000	10.000	0.250	42.54	234.00	97.15
11	5.000	0.000	0.250	39.02	73.07	85.14
12	5.000	20.000	0.250	23.88	255.29	96.85
13	5.000	10.000	0.000	48.53	126.06	75.13
14	5.000	10.000	0.500	21.65	110.72	96.12
15	5.000	10.000	0.250	44.69	150.58	82.13
16	5.000	10.000	0.250	42.68	150.72	86.75
17	5.000	10.000	0.250	42.91	150.91	89.60
18	5.000	10.000	0.250	42.33	150.73	83.66
19	5.000	10.000	0.250	42.10	150.69	81.24
20	5.000	10.000	0.250	42.22	150.85	87.30

**Table 2:** Regression coefficients, R^2^, adjusted R^2^, and lack of fit for the final reduced models.

Regression Coefficient	Creaming Index at 25 °C for 24 h (%) (Y_1_)	Viscosity at 25 °C for 24 h (η, mPa) (Y_2_)	Encapsulation Efficiency at 25 °C for 24 h (EE, %) (Y_3_)
β_0_	156.0566	−221.3047	77.3175
β_1_	26.7081	60.8535	−0.8330
β_2_	10.9112	26.3832	−1.6132
β_3_	59.7950	710.9080	39.4127
β_11_	0.3055	1.3495	−0.0852
β_22_	8.8685	−97.3024	−3.7376
β_33_	3.2432	−0.8407	1.1151
β_12_	1.9848	−3.7430	0.3852
β_13_	0.3795	−1.1750	0.1249
β_23_	123.5204	−517.9632	8.0626
R^2^	0.8729	0.9808	0.8654
R^2^ (adj)	0.6982	0.9545	0.6804
Regression (*p*-value)	0.0153	<0.0001	0.0187
Lack of fit (F-value)	167.2824	37.2226	2.2521
Lack of fit (*p*-value)	<0.0001	<0.0001	0.2001

β_i_, The estimated regression coefficients for the main linear effects. β_ii_, the estimated regression coefficient for the quadratic effects. β_ij_, the estimated regression coefficient for the interaction effects. _1_, SPI; _2_, MD; _3_, PGA.

**Table 3:** Experimental design of composite powder prepared by SPI, MD, and PGA with different contents.

Run Number	Independent Variables (%, *w*/*w*)		
	SPI (X_1_)	MD (X_2_)	PGA (X_3_)
1	2.113	4.227	0.106
2	7.887	4.227	0.106
3	2.113	15.774	0.106
4	7.887	15.774	0.106
5	2.113	4.226	0.395
6	7.887	4.227	0.395
7	2.113	15.774	0.395
8	7.887	15.774	0.395
9	0.000	10.000	0.250
10	10.000	10.000	0.250
11	5.000	0.000	0.250
12	5.000	20.000	0.250
13	5.000	10.000	0.000
14	5.000	10.000	0.500
15	5.000	10.000	0.250
16	5.000	10.000	0.250
17	5.000	10.000	0.250
18	5.000	10.000	0.250
19	5.000	10.000	0.250
20	5.000	10.000	0.250

## Data Availability

Data are contained within the article.
